# Phillyrin Prevents Ovariectomy-Induced Osteolysis by Inhibiting Osteoclast Differentiation

**DOI:** 10.1155/2022/6065494

**Published:** 2022-06-10

**Authors:** Geng Zhang, Ying Zhang, Cong Wang, Chenhe Zhou, Shigui Yan

**Affiliations:** ^1^Department of Orthopedic Surgery, Second Affiliated Hospital, School of Medicine, Zhejiang University, Hangzhou, China; ^2^Department of Orthopedic Surgery, Taizhou Hospital of Traditional Chinese Medicine, Taizhou, China

## Abstract

Postmenopausal osteoporosis is a metabolic disease caused by an imbalance between osteoclasts and osteoblasts. At present, the drug strategy for treating postmenopausal osteoporosis has some limitations and is unable to satisfy the demands of patients. *Phillyrin (Phil)* is an herbal extract from *Forsythiae Fructus*, with an inhibitory effect on osteolysis. In this study, we described the role of *Phil* in ovariectomy-induced osteoporosis and its effect on osteoclast differentiation *in vitro*. Eighteen female C57BL/6 mice were randomly divided into three groups: sham group (sham surgery and injection with 0.9% normal saline), ovariectomized group (ovariectomy and injection with 0.9% normal saline), and *Phil* group (ovariectomy and injecting *Phil* with 100 mg/kg for 2 days). Mice were sacrificed after 6-week *Phil* administration and femurs were harvested for microcomputed tomography (micro-CT) and histomorphology analyses. In vitro, we used different concentrations of *Phil* to study its effect on osteoclastogenesis. The results showed that the BV/TV, Tb.Th, and Tb.N in trabecular bone were increased in the *Phil* group compared with the OVX group, and the trabecular bone mass was remarkably decreased in the OVX group compared with the sham group. The number of osteoclasts was increased in the OVX group compared to the sham group, and the number and area of osteoclasts were decreased in the *Phil* group compared to the control group. Compared with the OVX group, the number and area of osteoclasts were reduced in the *Phil* group. In conclusion, *Phil* could inhibit the formation of osteoclasts, promote the growth of bone trabecular, and relieve osteoporosis caused by ovariectomy, with a certain clinical adoption value.

## 1. Introduction

Osteoporosis is a metabolic bone disease by the increased risk of fragility fractures [1], which is a major health problem worldwide. Presently, there are 10 million patients with osteoporosis in the United States and 27.6 million in Europe. In China, 11.7% of patients suffer from osteoporosis, most of whom are postmenopausal women and the elderly [2]. Osteoporosis can cause pain in the waist and lower back, which results in shortened body length and hunchback. It can even cause fractures, limb dysfunction, and death [3]. Osteoporosis not only leads to low quality of life [4] but also brings a heavy financial burden to patients and their families [5]. It is estimated that the annual expenditure of treating fragile osteoporotic fractures in the US by 2025 will increase by $25 billion [6] and that in China will be as high as 132 billion Yuan by 2035. Therefore, the prevention and treatment of osteoporosis are of great significance [7].

Currently, drugs used for the treatment of osteoporosis have their disadvantages and side effects to varying degrees. For example, the long-term application of menopausal hormone therapy drugs may cause cardiocerebral vascular events, thrombosis, endometrial cancer, and breast cancer [8]. The long-term use of bisphosphonates may lead to complications, such as mandibular necrosis [9]. Salmon calcitonin can increase the risk of tumors; however, its continuous use generally does not exceed 3 months [10, 11]. Selective estrogen receptor modulators, such as raloxifene, may also cause cardiovascular and cerebrovascular accidents as well as venous thrombosis [12]. Teriparatide has limitations of high cost, long duration of treatment, and late drug requirement [13]. Strontium ranelate may cause serious adverse cardiovascular and cerebrovascular reactions, including drug eruptions with eosinophilia and systemic symptoms [14, 15]. Therefore, a drug with fewer side effects is expected to inhibit osteoclast differentiation and improve osteogenesis. *Forsythiae Fructus*, a Chinese herbal medicine, was proposed.

*Forsythiae Fructus* is a commonly used herb in traditional Chinese medicine. It has a substantial curative effect on the treatment of fever, carbuncle, gonorrhea, inflammation, and erysipelas [16]. *Phillyrin* (*Phil*) is a lignan extracted from the dried fruit of *Forsythiae Fructus*, which has several pharmacological effects. It includes antioxidation, antiobesity, antivirus, anti-inflammation, and antipyretic effects. Besides, *Phil'*s melting point is between 184°C and 185°C, and it is the crystalline powder [17, 18]. According to the clinical study, *Phil* has an inhibitory effect on osteolysis of lipopolysaccharide (LPS) rat skulls [19]. However, there are few clinically relevant studies.

To further explore whether *Phil* could affect osteolysis and alleviate or treat osteoporosis, *Phil* was adopted on the ovariectomized (OVX) mice to evaluate the changes of bone mineral density in the mice, thus exploring the role of *Phil* in OVX-induced osteoporosis. It was hoped to provide effective treatment methods for patients with osteoporosis clinically.

## 2. Materials and Methods

### 2.1. Animal and Grouping

Eighteen healthy female C57BL/6 mice (10 weeks old and weighing approximately 20 g) were obtained from Slac Laboratory Animal (Shanghai, China). Mice were raised under specific pathogen-free conditions (20–25°C, 60% humidity, 12/12 h light/dark) and given free access to water and food. The animal experiments were approved by the Animal Care and Use Committee of Zhejiang University following the Guide for the Care and Use of Laboratory Animals published by the United States National Institutes of Health (NIH).

Mice were randomly divided into three groups: sham group (*n* = 6; sham operation with 0.9% normal saline (NS)), OVX group (*n* = 6; OVX mice administered with 0.9% NS), and *Phil* group (*n* = 6; OVX mice administered with *Phil* (Changsha Heking Biotechnology Co., Ltd., China) at a dose of 100 mg/kg every other day).

### 2.2. Surgical Procedure

Animals were anesthetized with an intraperitoneal injection of 5 mg/kg pentobarbital sodium (Sigma-Aldrich, Saint Louis, MO, USA) and fixed on the operating table in a prone position. An incision was made over the bilateral scapular line of the spine near the lower edge of the ribs under aseptic conditions to expose the retroperitoneal tissue. Then, the deep pink granulous ovarian tissue was detected on both sides, the fallopian tubes and distal ovarian vessels were ligated, and the ovaries were removed. After cleaning the wound, the muscle, fascial layers, and skin were closed. Finally, buprenorphine was injected to reduce pain, and the mice were placed in the prone or lateral position in a breeding box to allow for recovery. For the sham controls, the incision was closed after the aforementioned procedures without any further intervention. Simultaneously, the experimental groups received intraperitoneal *Phil* (Aladdin, Los Angeles, CA, USA) every other day from day 3 after the operation. Mice were sacrificed after 6 weeks, and femurs were fixed with 4% paraformaldehyde (BOSTER, Wuhan, China) and then stored at 4°C with 70% ethanol until use.

### 2.3. Microcomputed Tomography (Micro-CT) Scanning

The femurs of mice in the three groups were scanned by using the Scanco*μ*CT100 scanner (Scanco Medical AG, Bassersdorf, Switzerland). The scan parameters were as follows. The X-ray energy was set to −70 kV and 200 mA, the exposure time was −300 ms, and the region of interest was set to 10 *μ*m around the metaphysis of the talus. According to the two-dimension data, the cone-beam reconstruction software (SkyScan) was adopted to reconstruct CT images into three-dimensional images. From the three-dimensional images, the ratio of bone volume to tissue volume (BV/TV), the structural model index, trabecular number (Tb.N), trabecular thickness (Tb.Th), and trabecular separation (Tb.Sp) were measured and analyzed.

### 2.4. Histological Analysis

For histological processing, right femurs (*n* = 6/group) were subjected to decalcification in 10% ethylenediaminetetraacetic acid (EDTA) for 21 days and then embedded in paraffin. The samples were sectioned, mounted on glass slides, deparaffinized in dimethyl benzene, dealkylated in xylene, soaked in alcohol with a reduced concentration gradient, and finally immersed in distilled water for staining experiment. Sections were subjected to tartrate-resistant acid phosphatase (TRAP) (Sigma-Aldrich, Saint Louis, MO, USA) and hematoxylin and eosin (H&E) (Sigma-Aldrich, Saint Louis, MO, USA) staining following the manufacturer's protocol and then imaged under a light microscope (Olympus BX51, Tokyo, Japan). The histomorphometric parameters, including trabecular BV/TV, the number of TRAP + osteoclasts normalized to the bone area, the percentage of osteoclast surface per bone surface (OcS/BS, %), and the number of osteoclasts per unit bone perimeter (N.Oc/BS, mm), were calculated using the ImageJ software (NIH, Bethesda, MD, USA).

### 2.5. *In Vitro* Osteoclast Differentiation

Primary bone marrow monocytes were isolated and cultured in a complete medium (alpha-modification of Eagle's medium supplemented with 10% fetal bovine serum (Gibco-BRL, Sydney, Australia), 1% Penicillin-Streptomycin Liquid (KeyGEN, Nanjing, China)) with 25 ng/mL macrophage colony-stimulating factor (M-CSF) (R&D systems, Minneapolis, MN, USA) at 5% CO_2_ and 37°C until macrophages formed. Macrophages were inoculated into the osteoclastogenic medium at a density of 8 × 103 cells/well in 96-well plates to induce osteoclast differentiation (complete medium supplemented with 25 ng/mL M-CSF and 25 ng/mL receptor activator for nuclear factor-*κ* B ligand (RANKL)) (R&D systems, Minneapolis, MN, USA) and were treated with different doses of *Phil* (5 and 10 *μ*M). Media were replaced every other day. After 4 days, the cells were fixed in 2.5% glutaraldehyde for 30 min and stained with a TRAP Staining Kit following the manufacturer's guidelines. The number of TRAP-stained cells having no less than five nuclei was counted using a light microscope, and the area of the cells was measured.

### 2.6. Statistical Analysis

All the data are presented as the mean ± SD of at least three independent tests. Statistical analysis was performed using one-way analysis of variance and followed by Tukey's post hoc analysis. Statistical significance was established at *P* < 0.05.

## 3. Results

### 3.1. *Phil* Prevented OVX-Induced Bone Loss in Trabecular Bone

**Figure 1:**
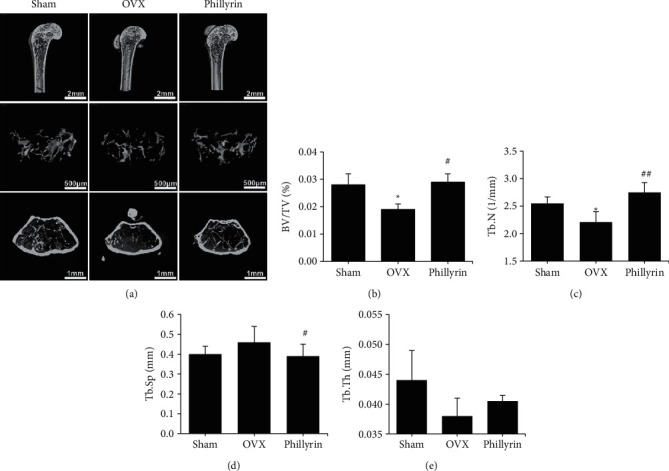
*Phillyrin* (*Phil*) prevented ovariectomized (OVX)-induced bone loss in trabecular bone. (a) Reconstruction of representative 3D microcomputed tomography (micro-CT) images of proximal femurs trabecular bone in each group. Scale bar = 2 mm, 500 *μ*m, and 1 mm. (b–e) The BV/TV, Tb.N, Tb.Sp, and Tb.Th values of the micro-CT data were analyzed from each sample. All values represent the mean ± SD (*n* = 6). ^*∗*^*P* < 0.05 and ^*∗∗*^*P* < 0.01*vs.* the Sham group. ^#^*P* < 0.05 and ^##^*P* < 0.01*vs.* the OVX group. BV/TV, bone volume to tissue volume ratio; Tb.N, number of trabeculae; Tb.Th, trabecular thickness; Tb.Sp, trabecular separation.

To study the role of *Phil* in preventing bone loss, a murine model of OVX-induced osteoporosis was established and treated with *Phil*. After a 6-week treatment, the proximal femurs were scanned using micro-CT. The results are shown in Figure 1. Compared with that of the sham group, the trabecular bone mass decreased in OVX groups. Moreover, this change was reversed in the drug-administered group. We observed an increase in the BV/TV, Tb.Th, and Tb.N in the trabecular bone of the *Phil* -treated group, compared with that of the OVX group.

**Figure 2:**
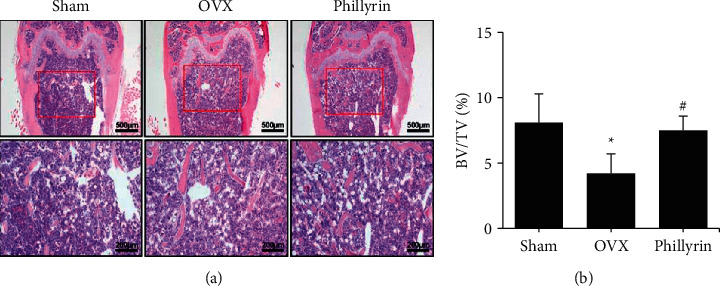
*Phillyrin* (*Phil*) prevented ovariectomized OVX-induced bone loss *in vivo*. (a) Histomorphological analysis was performed by hematoxylin and eosin (H&E) staining. Scale bar = 500 and 200 *μ*m. (b) The bone volume to tissue volume ratio (BV/TV) of histomorphological sections was measured and quantified. All values represent the mean ± SD (*n* = 6). ^*∗*^*P* < 0.05 and ^*∗∗*^*P* < 0.01*vs.* the Sham group. ^#^*P* < 0.05 and ^##^*P* < 0.01*vs.* the OVX group.

To further verify the protective functions of *Phil* against trabecular bone loss, the right femurs were decalcified and assessed by histomorphology. H&E staining (Figure 2) showed that the femoral trabecular mass in the OVX group was observably reduced, compared with that in the sham operation group; whereas, the mitigative effect could be observed in the *Phil*-treated groups, which was consistent with the micro-CT scan.

### 3.2. *Phil* Inhibited Osteoclastogenesis *In Vivo*

**Figure 3:**
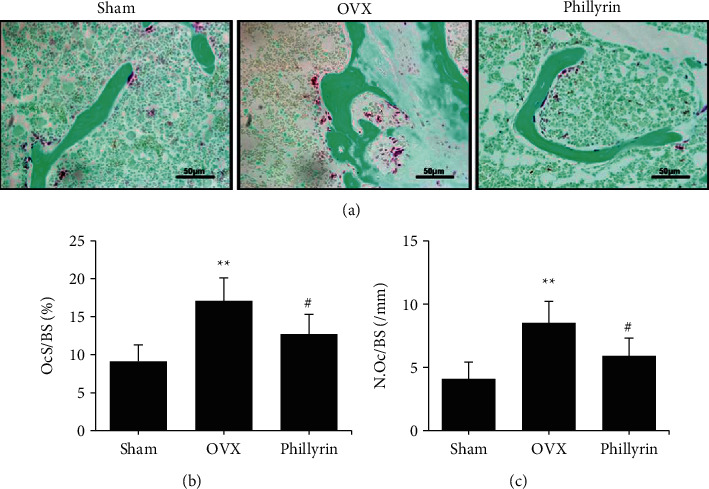
*Phillyrin* (*Phil*) inhibited osteoclastogenesis *in vivo*. (a) Tartrate-resistant acid phosphatase (TRAP) staining was performed on decalcified parts of distal femurs. Scale bar = 50 *μ*m. (b, c) The OcS/BS and N.Oc/BS values were measured with TRAP-stained sections. All values represent the mean ± SD (*n* = 6). ^*∗*^*P* < 0.05 and ^*∗∗*^*P* < 0.01*vs.* the Sham group. ^#^*P* < 0.05 and ^##^*P* < 0.01*vs.* the OVX group.

Considering the important role that osteoclasts play in osteolysis, we evaluated whether *Phil* exerted an inhibitory effect on osteoclastogenesis *in vivo*.  Figure 3 illustrates that compared with that in the sham group, an increased number of osteoclasts was found in the OVX group. *Phil* injection significantly reduced the OVX-induced increase in the number and size of osteoclasts.

OcS/BS, percentage of osteoclast surface per bone surface; N.Oc/BS, number of osteoclasts per unit bone circumference.

### 3.3. *Phil* Attenuated Osteoclast Differentiation *In Vitro*

**Figure 4:**
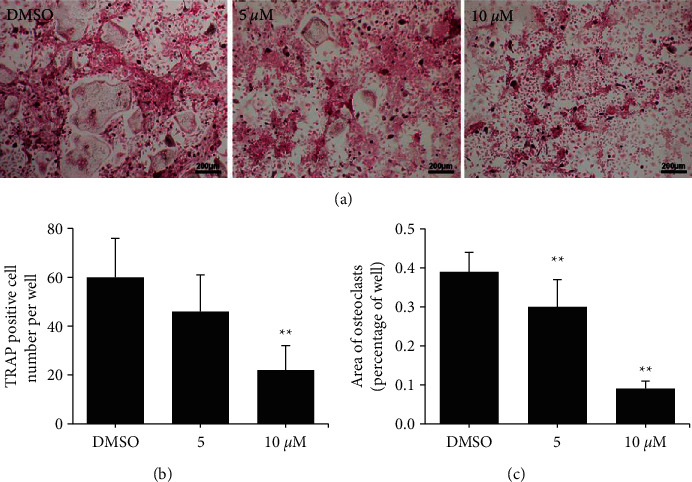
*Phillyrin* (*Phil*) attenuated osteoclast differentiation *in vitro*. (a) Representative tartrate-resistant acid phosphatase (TRAP) staining images from bone marrow macrophages (BMMs) treated with dimethyl sulfoxide (DMSO) or different doses of *Phil* in the presence of receptor activator for nuclear factor-*κ* B ligand (RANKL) and macrophage colony-stimulating factor (M-CSF) for 4 days. Scale bar = 200 *μ*m. (b, c) The area of TRAP-positive multinuclear osteoclasts were measured and the number was quantified (≥3 nuclei) in each well of the 96-well plate. All values represent the mean ± SD. ^*∗*^*P* < 0.05 and ^*∗∗*^*P* < 0.01.

To examine whether *Phil* has an attenuation effect on osteoclast differentiation, primary bone marrow monocytes were isolated and investigated *in vitro*. In Figure 4, the number and area of osteoclasts were reduced in the *Phil* group compared with the OVX group. Moreover, at higher doses of *Phil*, less number of osteoclasts was observed. These results demonstrated that *Phil* attenuated osteoclast differentiation in a dose-dependent manner *in vitro*.

## 4. Discussion

Osteoporosis, which affects millions of people worldwide, is an enormous and growing public health challenge. Currently, many drugs and methods for the clinical treatment of osteoporosis are available, such as estrogen replacement therapy and bisphosphonates. However, the lack of clinical evidence supporting their long-term therapeutic effect and the possibility of adverse reactions keep many patients, who may profit from these drug therapies, from taking the drugs [20]. In this study, we proved that *Phil* protects against bone loss caused by estrogen deficiency by inhibiting the differentiation of osteoclasts. Therefore, we believe that *Phil* might be a potential drug for the treatment of osteoporosis.

Animal models for osteoporosis include disuse osteoporosis, glucocorticoid-induced osteoporosis, and postmenopausal osteoporosis [21]. An OVX animal is a typical experimental model of postmenopausal osteoporosis caused by estrogen deficiency in which the mice model mimics humans in terms of estrogen deficiency-induced postmenopausal osteoporosis. Therefore, the model is widely used to evaluate and develop new drugs for postmenopausal osteoporosis [22]. Previous studies have shown that osteoporosis in mice due to estrogen deficiency is always accompanied by weight gain and uterine weight loss [23]. This phenomenon was also observed in our experiment, which indicates that the OVX surgery was effective and that the model was successfully established.

*F. suspensa* (Thunb.) Vahl (Oleaceae) is a traditional herbal medicine that has been widely employed for the clinical treatment of several infectious diseases [24]. Furthermore, it is widely applied in the food and cosmetic industries as a component of our daily lives [25, 26]. Moreover, previous experimental studies have reported the therapeutic effect of *Phil* in certain diseases. For example, *Phil* can be developed as a therapeutic agent to treat influenza A virus infection [27], improve glucose and lipid metabolism abnormalities in obese patients [28], and play a protective role in LPS-induced osteolysis [19]. In our study, both micro-CT and H&E staining results showed that the bone mass was decreased, and the number of osteoclasts was increased in the TRAP-stained histological sections from the OVX groups. After the drug intervention, a dose-dependent preventive effect was found. Micro-CT and H&E staining results showed that the dose (100 mg/kg for 2 days) in the experiment significantly inhibited the bone loss in the OVX groups. Simultaneously, TRAP staining showed that a decreased number of osteoclasts in the OVX groups. Moreover, *in vitro* experiments demonstrated that the application of this drug could suppress the differentiation of osteoclasts in a concentration-dependent manner. Therefore, our results suggested that *Phil* could attenuate OVX-induced bone loss *in vivo* and osteoclast differentiation *in vitro* in a concentration-dependent manner.

In summary, this study indicates that *Phil* inhibits OVX-induced osteolysis by inhibiting osteoclast differentiation. Our results demonstrated that *Phil* might be a novel and effective anti-osteoporosis agent.

## 5. Conclusion

*Phil* was applied for the ovariectomized (OVX) mice to evaluate changes in bone mineral density and explore the role of *Phil* in osteoporosis induced by OVX. The results showed that *Phil* was helpful to inhibit the formation of osteoclasts, promote the growth of bone trabecular, and relieve osteoporosis caused by ovariectomy, with the value of clinical adoption. However, this experiment uses animal models as the research objects, so it needs to be further explored in the clinic before the clinical adoption. Furthermore, through this experiment, *Phil* can effectively inhibit bone loss, with good development prospects.

## Data Availability

The data used to support the findings of this study are available from the corresponding author upon request.
